# From Spectral Maps to Biomedical Evidence: A Minimum Evidentiary Framework for Label‐Free Raman Imaging (Reply to Letter‐to‐the‐Editor)

**DOI:** 10.1002/advs.77930

**Published:** 2026-09-27

**Authors:** Jiaxin Li, Xiaoyu Zhao, Jun Yu

**Affiliations:** ^1^ Institute of Reproductive Medicine Medical School Nantong University Nantong China; ^2^ SuperVision Medicine Co., Ltd. Wuxi China

Dear Editor,

Thank you for informing us of the reader's correspondence and for giving us the opportunity to clarify the analytical basis, scope, and reproducibility of our published study. We always welcome normal, constructive academic exchange among scientists, including discussions with experts in Raman spectroscopy, reproductive medicine, and related interdisciplinary fields. Such scientific dialogue is essential for improving methodological transparency, clarifying interpretations, and advancing the collective understanding of emerging technologies. We have reviewed the Correspondence carefully, together with the published article, the original peer‐review record, the Supporting Information, the previously published qRamanomics methodology and code in Cell Reports Methods 2023, and the underlying Raman input/output files.

We agree with the general principle that the evidentiary strength of a Raman‐derived result should match the strength of the claim. We respectfully disagree, however, with several central inferences in the Correspondence because they evaluate our study as if it claimed absolute concentration measurements and definitive independent chemical identification of 42 analytes. The published article explicitly states that the NNLS‐derived maps are reference‐constrained relative distributions rather than absolute molecular concentration maps, and the Discussion explicitly describes the small‐molecule maps as Raman‐inferred/model‐constrained representations rather than definitive molecular identities for every analyte.

We also identify several reporting details that can and should be clarified. We provide those clarifications below and supply additional model diagnostics, raw‐data examples, and reference‐library files. In our view, these additions address reproducibility and transparency without changing the underlying data or the principal biological observations.

We also noted several potentially misleading elements in the figure provided by this reader of the Correspondence: The illustrated microscope does not correspond to any confocal Raman microscope and appears to be a generic microscopy schematic. Likewise, the spectral traces shown in the figures are schematic illustrations rather than experimentally acquired Raman spectra. This distinction is important because these graphical elements should not be interpreted as representative Raman measurements or as evidence concerning the analytical performance of our SLRI platform. In the figures, the tissue image appears to be a conventional histology‐style image rather than a Raman‐derived molecular image, and the microscope illustration is again a generic schematic rather than the Raman instrument used in our work. The blue‐colored optical beam shown in this schematic also does not correspond to the 532‐nm excitation used in our experiments; the colour of the laser light should be green instead.

Executive summary

**Table:** 

Issue	Our position/evidence
Analytical claim	The study reports reference‐constrained NNLS spectral contribution maps. It does not report 42 absolute molecular concentration maps.
Imaging modality	SLRI is spontaneous confocal hyperspectral Raman imaging with single‐beam 532 nm excitation and downstream NNLS. The article states this explicitly. It is not stimulated Raman scattering (SRS).
Code and data lineage	The MATLAB qRamanomics processing/NNLS implementation was previously published and archived (https://doi.org/10.5281/zenodo.7628912). The same processing/NNLS codebase was used here with a study‐specific 42‐reference library. We now provide the normalized reference spectra, interpolation axis, representative raw hyperspectral data, and a corresponding coefficient‐map/TIFF pair.
New identifiability diagnostics	The 42‐reference design matrix is full rank (42/42). Pairwise correlations and clustering reveal the calculated spectral similarity among some related references. The 2‐norm condition number is 391.5 for the actual unit‐area‐normalized design matrix (242.6 after additional L2 column normalization as a diagnostic). These results support transparent, model‐constrained interpretation rather than a claim that all 42 spectra are orthogonal.
Sample design	In this study, SLRI was applied to representative testes from each experimental group to resolve in situ spatial biochemical features associated with established biological events. The corresponding phenotypes were independently validated by immunofluorescence, genetic, and functional assays with consistent results. Therefore, SLRI was used for spatial biochemical characterization rather than population‐level statistical inference.
Sample state and stability	Raman‐imaged testes were fixed in 4% PFA for 20 min and washed three times in PBS before agarose/PBS imaging. Thus, long scans did not involve living‐tissue motion or ongoing metabolism. From the published figure and the raw Raman data provided, all spectra have a high signal‐to‐noise ratio and all images are clear.
Correspondence figure and AI	The Correspondence Figure provided by this reader is explicitly conceptual; its microscope icon and spectral graphics are illustrative rather than experimental Raman data. Such a schematic is not evidence that an AI‐centric alternative is analytically superior. AI may be useful in Raman research, but it is neither necessary nor evidentiary for validating this deterministic NNLS study. The microscope in the Figures (provided by this reader) is clearly not a Raman microscope, and the colour of the laser should be green for 532 nm, not blue. This core point, we believe, should be the most fundamental cognitive core for Raman experts.

## 1 Scope of the SLRI Study and Biological Replication

The Correspondence raises questions regarding biological replication and the interpretation of SLRI imaging data. We clarify that the primary objective of this study was to establish a high‐dimensional, label‐free Raman imaging framework for resolving the spatial organization of biochemical components within intact tissues, rather than to perform population‐level statistical comparisons or determine absolute molecular concentrations. Given the technical characteristics of three‐dimensional Raman imaging, including prolonged acquisition time, large hyperspectral data volume, and high‐resolution volumetric reconstruction, SLRI was applied as a spatial mapping approach to characterize biochemical distributions and their remodeling during specific biological processes.

The biological phenotypes defining this experimental system were not inferred solely from SLRI imaging but were supported by extensive previous observations and independent validations. Using two independent *Cyp4ae1* RNAi lines targeting different regions of the gene, we consistently observed the *Cyp4ae1* deficiency‐induced spermatogonial differentiation arrest phenotype. In addition, LD540 and TOM20 staining assays, ATP and NADP+/NADPH assays, and non‐targeted metabolomic analyses further supported *Cyp4ae1* knockdown‐associated alterations, including lipid accumulation and mitochondrial abnormalities. Therefore, SLRI images should be interpreted as high‐dimensional spatial biochemical characterization embedded within a broader replicated biological study, rather than as an independent population‐level quantitative omics dataset.

Representative‐sample strategies combined with volumetric Raman acquisition have been widely applied in label‐free Raman imaging studies focusing on in situ biochemical organization and molecular distribution within intact biological specimens. Consistent with these approaches, our study uses SLRI primarily as a spatial biochemical imaging strategy to resolve tissue‐level molecular organization and remodeling, rather than as a population‐level quantitative omics platform.

In the original Statistics section, we used the phrase “relative concentrations” for SLRI violin plots. Because the SLRI image datasets were not designed as population‐level replication, we do not rely on pixel‐level t‐tests as evidence of biological replication.

## 2 Quantitative Meaning of the Maps (Correspondence Lines 59–74)

The Correspondence states that an NNLS coefficient is not, by definition, an absolute molecular concentration. Our article makes the same distinction; we have never stated our results are absolute molecular concentrations. Section 5.7 states that the NNLS‐derived maps represent relative abundance distributions constrained by the reference spectral library rather than absolute molecular concentration maps. The Discussion further states that the Raman‐inferred features are not independently validated as definitive identities for every individual metabolite.

The appropriate analytical hierarchy is therefore: measured Raman spectrum → deterministic reference‐constrained NNLS coefficient → spatial coefficient map. The current *Drosophila* study does not convert these coefficients to mg/mL or mol/L. The earlier qRamanomics study established a separate concentration‐calibrated implementation for selected major biomolecular classes; that calibration is not claimed for all 42 *Drosophila* references.

Regarding the concern that Supplementary Figure presents quantitative distributions for only three of the 42 reference components (UFA, SFA, and Cyt C (ox)), we emphasize that these three components were selected as representative examples for quantitative comparison between Stage 1 and Stage 2, rather than indicating that SLRI was limited to only three molecular components. The manuscript has explicitly stated that “the quantitative analysis of UFA, SFA, and Cyt C (ox) reflects the overall signal intensity changes across the entire testis sample, but may not fully capture local differences within specific structures.” Therefore, these quantitative analyses were intended to illustrate representative biochemical signal trends at the whole‐testis level, whereas the major biological interpretations were based on the spatial distribution and remodeling patterns resolved by SLRI. The violin plots were generated from NNLS‐derived Raman signal intensity distributions at the pixel level within each individual testis and were used to visualize the relative distribution of Raman‐derived biochemical signals under the same acquisition and analytical conditions, rather than to represent independent biological replicates. As clarified above, pixels within a single specimen are spatially correlated measurements and were not considered independent biological replicates for statistical inference.

Consequently, criticism based on the absence of analyte‐specific absolute concentration calibration should not be used to invalidate a claim that was explicitly framed as relative, model‐constrained spatial spectral inference. Where the published text uses shorthand such as “enriched,” the intended meaning is a relatively higher distribution of the corresponding reference‐constrained component, not a calibrated concentration increase. This interpretation is consistent with the established principles of Raman spectroscopy.

## 3 Data Lineage, Code, and Reconstructibility (Correspondence Lines 75–93)

The Correspondence states that the computational workflow is not reconstructible from the public record. We agree that the Advanced Science article could have linked the computational implementation and study‐specific data more explicitly. However, the spectral‐processing/NNLS implementation was not newly invented for this paper. The same MATLAB qRamanomics processing and NNLS codebase was used, with the study‐specific reference library supplied as the principal changed analytical input. The original qRamanomics code was deposited publicly with the 2023 paper and remains archived through Zenodo (https://doi.org/10.5281/zenodo.7628912). Similar methods have been used for decades.

For this Response, we provide the following study‐specific files to make the data lineage explicit:

1. Normalized Reference Raman Spectra.zip — the 42 reference spectra actually used in the *Drosophila* analysis.
2. Stage1‐3D_layer1.mat — a representative raw hyperspectral Z‐plane containing 30,771 measured spectra on a 263 × 117 XY grid, each with 1,114 Raman‐shift channels. This spatial size exactly matches the Stage 1 3D acquisition reported in the Supplementary Table.
3. Cyt C (ox)‐stage1_layer1.mat — the numerical post‐NNLS Cyt C coefficient vector for a Stage 1 2D map.
4. Cyt C (ox)‐stage1_layer1.mat.tiff — the corresponding rendered TIFF. Reshaping the numerical coefficient vector to 600 × 686 reproduces the TIFF with Pearson r ≈ 0.9999, demonstrating the direct numerical map‐to‐image lineage.
5. 42_reference_library_diagnostics.xlsx and associated figures — pairwise correlations, condition‐number/SVD analysis, and hierarchical clustering.

The representative Stage1‐3D layer demonstrates that the underlying data are not merely pseudocolor images: the file contains the full pixel‐resolved spectral matrix. The corresponding coefficient file/TIFF pair demonstrates the subsequent transformation from numerical NNLS output to the displayed image.

## 4 Reference‐Spectrum Normalization and Alignment

The correct 42‐reference library contains 42 spectra, each sampled on the same 1,134‐point Raman‐shift axis (approximately 0.81–3849.72 cm^−^^1^). The reference spectra were normalized to equal integrated spectral area. Before fitting a tissue dataset, the normalized references were linearly interpolated onto the measured Raman‐shift axis of that dataset. For the representative Stage 1 Z‐plane supplied here, the measured axis contains 1,114 points spanning approximately 3.88–3797.81 cm^−^^1^; this range lies within the reference range, so no spectral extrapolation is required.

This normalization is important for interpretation. Equal‐area reference scaling places the reference shapes on a common numerical basis but intentionally does not preserve compound‐specific absolute Raman scattering cross‐sections. Therefore, the fitted coefficients are interpreted as relative reference‐constrained spectral contributions, not molar abundance (we have never claimed in our paper that we derived the molar/absolute concentration abundance). Comparisons are most defensible for the same component across spatial regions/conditions, rather than as absolute cross‐molecule stoichiometry.

## 5 Identifiability and Collinearity of the 42‐Reference Model (Correspondence Lines 94–105)

We evaluated the exact 42‐reference library after interpolation to the representative measured Raman axis and application of the same fitting window used in the qRamanomics workflow (400–3100 cm^−^^1^, excluding the 1800–2700 cm^−^^1^ silent region).

**Table:** 

Diagnostic	Result
Reference components	42
Fitting variables	500 Raman‐shift points
Matrix rank	42/42
2‐norm condition number (actual unit‐area reference matrix)	391.5
2‐norm condition number after additional L2 column normalization (diagnostic only)	242.6

**FIGURE R1:**
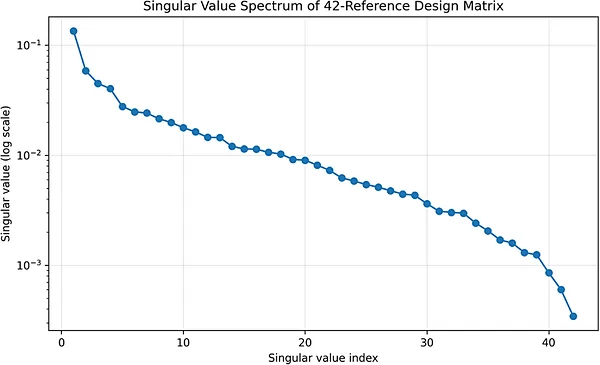
Singular‐value spectrum of the 42‐reference design matrix used for conditioning assessment.

**FIGURE R2:**
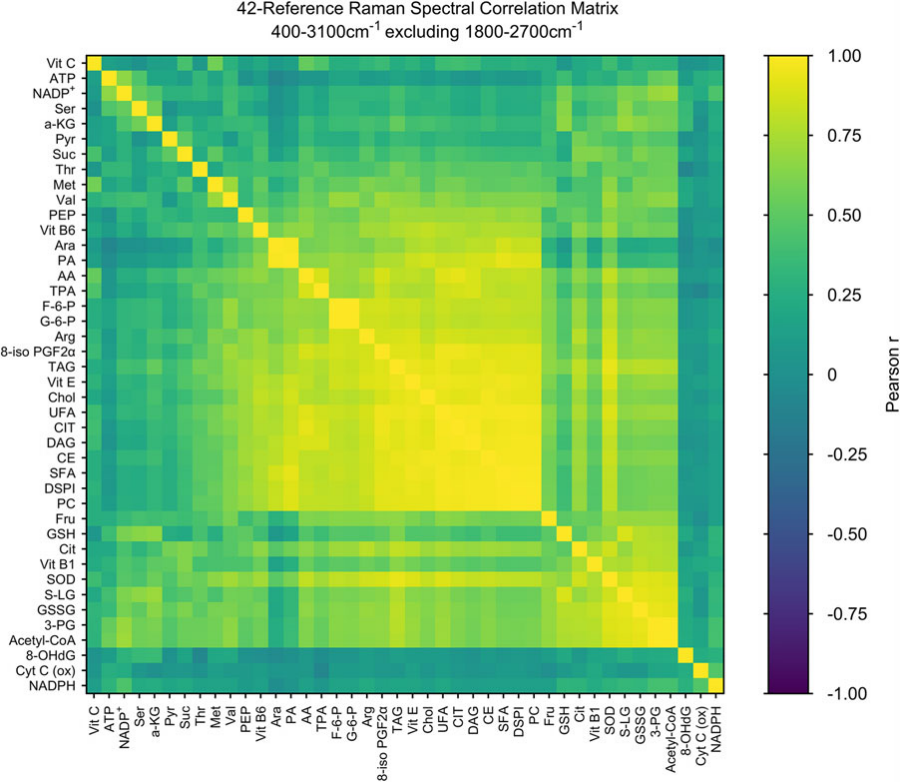
Cluster‐ordered Pearson correlation matrix of the 42 normalized reference Raman spectra after interpolation to the measured Raman axis and application of the fitting window.

**FIGURE R3:**
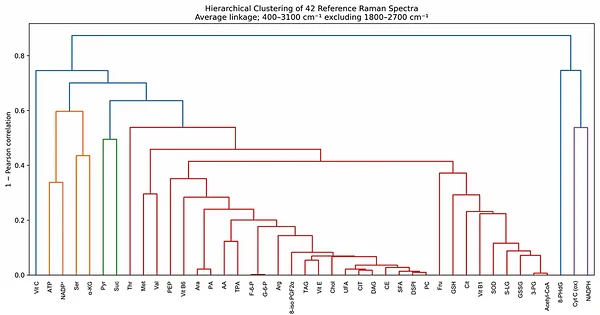
Hierarchical clustering of the 42 reference spectra using 1 − Pearson correlation as spectral distance and average linkage.

The matrix is full rank, demonstrating that there is no exact linear dependence among the 42 reference columns (Response Figure R1). At the same time, the condition number and pairwise correlation matrix reveal substantial collinearity among several chemically related references (Response Figures R2 and R3). We do not present these diagnostics as proof that all 42 components are mutually orthogonal or unconditionally identifiable; instead, they explicitly quantify the model dependence that motivates the “Raman‐inferred/reference‐constrained” terminology used in the paper. The new diagnostics therefore strengthen transparency while also defining the limits of the reference model.

We do not consider a noise‐free synthetic mixture generated and refitted with the identical design matrix to be meaningful validation by itself, because such a test would be mathematically circular.

## 6 Molecular Labels, Reference Standards, and Orthogonal Support (Correspondence Lines 106–138)

The Supplementary Table transparently reports the purified standards used to define the 42‐reference library. Accordingly, labels such as SFA, UFA, TAG mix, PC, or SOD were stated as reference‐associated spectral components/proxies defined by the stated standards, not as species‐complete chemical inventories.

The *Drosophila* study did not establish analyte‐specific tissue‐matrix LOD/LOQ, spike‐recovery, or definitive orthogonal localization for every reference. This limitation is already reflected in the published Discussion, which explicitly states that the maps are spectrally inferred/model‐constrained and not independently validated as definitive molecular identities for each metabolite.

The study nevertheless contains orthogonal biological support at the appropriate scale. ATP and NADP+/NADPH were measured independently in whole‐testis lysates; lipid droplets and mitochondrial organization were assessed by LD540 and TOM20; the differentiation phenotype was supported by Vasa/1B1, PH3/EdU, and an independent *Cyp4ae1* RNAi line; and non‐targeted metabolomics independently demonstrated genotype‐associated changes in lipid/lipid‐like and amino‐acid‐related metabolism. We agree that bulk assays do not prove microscopic localization of an individual Raman component, and we do not present them as such. They provide orthogonal support for the broader metabolic remodeling in which the spatial Raman‐inferred patterns are embedded.

## 7 Acquisition Duration, Fixation, Drift, and 3D Reconstruction (Correspondence Lines 152–164)

The Correspondence correctly notes that some large point‐scanned datasets require many hours of nominal integration time. The article, however, did not explicitly state the Raman‐specific fixation step. We clarify that the testes used for Raman imaging were fixed in 4% paraformaldehyde for 20 min, washed three times in PBS, embedded in agarose, and maintained in PBS during Raman acquisition. This fixation procedure represents a standard sample preparation step routinely used for *Drosophila* testis histological analyses and therefore was not specifically emphasized as an independent methodological parameter in the original article. The Raman specimens were therefore not living tissues undergoing cell migration, differentiation, active metabolite turnover, or morphological changes during the scan. Fixation substantially removes the biological‐motion scenario implied by treating the samples as potentially live/fresh.

The channel‐to‐channel molecular maps do not require registration of separately acquired modalities: all molecular coefficients at a given XY coordinate are derived from the same measured Raman spectrum. For 3D visualization, consecutive z‐planes were acquired continuously within the same microscope coordinate system and stacked according to the recorded XYZ coordinates. No intensity‐based inter‐channel registration is involved.

Moreover, the punctate/ring‐like patterns discussed in the article were identified directly in individual XY planes. Supplementary Figures display those patterns in 2D and compare measured spectra from peripheral punctate loci and central diffuse loci within the same RB. Thus, variation in z‐step size cannot generate an XY‐plane punctate pattern. The 3D rendering visualizes the already measured 2D coefficient maps rather than creating the underlying spectral heterogeneity.

## 8 Spontaneous Raman Modality and the SRS Citation Issue (Correspondence Lines 139–151)

There is no experimental ambiguity regarding the Raman modality. The article repeatedly describes the method as hyperspectral spontaneous Raman spectroscopy/confocal Raman microscopy, and the instrument description specifies a single 532 nm excitation beam, point scanning, full‐spectrum acquisition, EMCCD detection, and downstream NNLS. These are features of spontaneous confocal Raman microscopy, not stimulated Raman scattering.

Importantly, the Correspondence itself correctly reaches the same classification. Its criticism is therefore not that our experiment was actually SRS; rather, it points out that our Reference 45 is an SRS review placed after the Bio‐SV instrument sentence. The review discusses multiple Raman‐based technologies, including spontaneous Raman and stimulated Raman approaches; however, our study specifically employs spontaneous confocal Raman microscopy. The Bio‐SV spontaneous Raman microscope is one of the top 7 Raman brands in the world, and they provide top‐quality spontaneous and stimulated Raman microscopes; this is the reason why the Bio‐SV microscope provided by SuperVision Medicine Co., Ltd. was mentioned in the Nature Photonics Review in 2025.

## 9 The Correspondence Figure and its AI‐Centered Closing Argument

The latter part of the Correspondence proposes an AI‐ready multimodal framework and uses this figure as a conceptual illustration. We do not object to a conceptual schematic, but we do not consider it evidence that the proposed framework is analytically superior to, or a validation benchmark for, the SLRI study.

Specifically, the Correspondence Figure uses a generic microscope illustration rather than a documented Raman microscope, and the spectral/analytical graphics in these Figures are stylized illustrations rather than measured Raman spectra from an experimental benchmark. The figure legend itself describes panel C as a conceptual multimodal spatial‐biology paradigm. These elements are therefore pedagogical graphics, not validation data.

AI may be valuable for future Raman denoising, representation learning, multimodal integration, or prediction. However, AI is not necessary for the analytical validity of the present study, and it cannot substitute for an explicit auditable transformation from measured spectra to model output. For this study, reproducibility is grounded in a deterministic mathematical pipeline: fixed input spectra and metadata, a specified reference matrix, interpolation/preprocessing, and NNLS optimization. Given identical inputs and code, the numerical output is reproducible without learned‐model variability.

We therefore respectfully suggest that the editorial reliability question should be assessed on the actual SLRI data lineage, model diagnostics, and claim scope. The prospective AI discussion in the Correspondence is scientifically interesting but does not constitute evidence that the published SLRI results are unreliable or that an AI‐based alternative has superior analytical validity. We note that the Correspondence Figure (provided by this reader) appears to be a conceptual illustration rather than a data‐derived experimental figure. If AI‐assisted tools were used for figure generation, appropriate disclosure according to journal policies would be important. Regardless of the figure‐generation approach, the scientific evaluation should focus on whether the proposed framework provides experimentally validated evidence.

We welcome new ideas and frameworks, provided they are supported with key experimental data. We strongly encourage the reader to present one or two core datasets in the correspondence to substantiate their proposal. Such contributions would greatly benefit the Raman community.

## 10 Point‐by‐Point Classification of the Correspondence Lines 59–164

**Table:** 

Lines	Concern	Assessment	Assessment and response
59–65	NNLS coefficient is not an absolute concentration	Overstated/already addressed	Paper explicitly states relative reference‐constrained maps, not absolute concentration.
66–74	Only three components quantified; color scales/statistical units	Have already mentioned in the paper	The Correspondence misinterprets the purpose of the quantitative analysis: the three components were selected as representative examples, and SLRI interpretations are based on NNLS‐derived spatial distribution patterns rather than color intensity alone.
75–82	Raw data/code/library not auditable	Overstated	Public qRamanomics code exists; 42 references and acquisition table were published; now provide raw hyperspectral example, normalized reference library, x‐axis, coefficient file, and TIFF.
83–93	Preprocessing/reconstruction not reproducibly specified	Have already mentioned in the paper	Same published processing/NNLS codebase was used; provide exact code archive and study‐specific input files; clarify simple XYZ stacking.
94–105	42‐component identifiability not demonstrated	Have already mentioned in the paper	Provide 42×42 correlations, full rank, condition number, SVD, and clustering; offer residual/leave‐one‐out/cross‐talk tests.
106–114	Class labels exceed specificity of a single standard	Overstated/already addressed	Treat as reference‐associated spectral components/proxies; standards are transparently listed.
115–123	No analyte‐specific LOD/LOQ/spike recovery	Have already mentioned in the paper	No absolute concentration claim; paper already qualifies identities; orthogonal assays support broader biology, not pixel‐level identity.
124–138	Qualification is incompatible with metabolic conclusions	Overstated	Association‐level spatial remodeling can be supported by model‐constrained spectral patterns plus genetic/orthogonal biology; causal transfer claims were explicitly softened.
139–151	Spontaneous Raman vs SRS	Overstated/already addressed	Our study identifies itself as spontaneous Raman. The author never mentioned Stimulated Raman. This Review listed SuperVision Medicine as one of the seven top global Raman microscope providers.
152–164	Long scans undermine spatial features	Overstated/already addressed	Samples were PFA‐fixed; distinguish biological motion from instrumental drift. XY puncta are present in individual planes and do not depend on z reconstruction.

## 11 Conclusion and Request to the Editor

We take the reliability question seriously and agree that transparent data lineage and model diagnostics are important. However, we have actually provided the full datasets during the review process before our paper was published. We have stated that the data were available upon request and have never received emails asking for our data. Due to a clearance issue, we here provide the link to the data again, although we have already provided (https://github.com/supervision‐xyz/SLRI‐Fly‐testis/branches).

The supplied materials show that the study is based on real pixel‐resolved hyperspectral Raman data, a documented 42‐reference library, deterministic interpolation/preprocessing, and a previously published NNLS implementation. The reference matrix is full rank, while the new correlation/condition/clustering analyses transparently identify the expected collinearity among some related references. This is precisely why the published paper qualifies the resulting maps as Raman‐inferred, model‐constrained distributions rather than absolute concentrations or definitive identities for every analyte.

We therefore respectfully disagree that the concerns presented in the Correspondence establish unreliability of the published findings. We appreciate the Editor's opportunity to respond to the Correspondence. The clarifications and supporting information provided above demonstrate the methodological rationale and analytical basis underlying our study. We hope that this response helps to accurately contextualize the workflow and conclusions presented in our original article.

## Appendix A. Supporting materials supplied with this Response

**Table:** 

Material	Purpose
Normalized rReference Raman sSpectra	Correct normalized 42‐reference library used for SLRI.
Stage1‐3D_layer1.mat	Representative raw Stage 1 3D Z‐plane: 263 × 117 spatial pixels, 1,114 Raman‐shift channels.
Cyt C (ox)‐stage1_layer1.mat	Numerical Cyt C (ox) NNLS coefficient vector for the corresponding Stage 1 2D map.
Cyt C (ox)‐stage1_layer1.mat.tiff	Rendered Cyt C (ox) map corresponding to the coefficient vector.
42_reference_library_diagnostics.xlsx	Correlation matrix, ranked pairwise correlations, condition‐number/SVD summary, cluster order, and singular values.
singular_value_spectrum.png	Response Figure R1.
correlation_matrix_clustered.png	Response Figure R2.
hierarchical_cluster_dendrogram.png	Response Figure R3.
qRamanomics code archive	Previously published deterministic MATLAB implementation: Zenodo (https://doi.org/10.5281/zenodo.7628912).

**Appendix B. References cited in this Response**
